# The Mechanism of Pseudomorphic Transformation of Spherical Silica Gel into MCM-41 Studied by PFG NMR Diffusometry

**DOI:** 10.3390/ma6093688

**Published:** 2013-08-26

**Authors:** Wolf-Dietrich Einicke, Dirk Enke, Muslim Dvoyashkin, Rustem Valiullin, Roger Gläser

**Affiliations:** 1 Institute of Chemical Technology, Universität Leipzig, Linnéstr. 3, 04103 Leipzig, Germany; E-Mails: wolf-dietrich.einicke@uni-leipzig.de (W.-D.E.); dirk.enke@uni-leipzig.de (D.E.); 2 Institute of Experimental Physics I, Universität Leipzig, Linnéstr. 5, 04103 Leipzig, Germany; E-Mails: muslimd@chem.ufl.edu (M.D.); valiullin@uni-leipzig.de (R.V.)

**Keywords:** pseudomorphic transformation, silica gel, LiChrospher^®^ Si 60, MCM-41 spheres, PFG NMR, diffusion

## Abstract

The pseudomorphic transformation of spherical silica gel (LiChrospher^®^ Si 60) into MCM-41 was achieved by treatment at 383 K for 24 h with an aqueous solution of cetyltrimethylammonium hydroxide (CTAOH) instead of hexadecyltrimethylammonium bromide (CTABr) and NaOH. The degree of transformation was varied via the ratio of CTAOH solution to initial silica gel rather than synthesis duration. The transformed samples were characterized by N_2_ sorption at 77 K, mercury intrusion porosimetry, X-ray diffraction (XRD) and scanning electron microscopy (SEM). Thus, MCM-41 spheres with diameters of *ca.* 12 μm, surface areas >1000 m^2^ g^−1^, pore volumes >1 cm^3^ g^−1^ and a sharp pore width distribution, adjustable between 3.2 and 4.5 nm, were obtained. A thorough pulsed field gradient nuclear magnetic resonance (PFG NMR) study shows that the diffusivity of n-heptane confined in the pores of the solids passes through a minimum with progressing transformation. The final product of pseudomorphic transformation to MCM-41 does not exhibit improved transport properties compared to the initial silica gel. Moreover, the PFG NMR results support that the transformation occurs via formation and subsequent growth of domains of <1 μm containing MCM-41 homogeneously distributed over the volume of the silica spheres.

## 1. Introduction

The discovery of the M41-S family of ordered mesoporous silicas [1] led to a large number of studies on potential applications of these materials in the fields of sorptive separation and heterogeneous catalysis. The use of these silicas in chromatographic systems or continuous catalytic processes requires careful control of the particle morphology and size [2]. In the last decade, considerable progress was made in tuning the spherical morphology during the synthesis of MCM-41 materials by controlled hydrolysis [3,4], modified Stöber synthesis [5,6] and spray drying [7,8]. However, the systematic control of particle morphology and size as well as the pore architecture within the particles is a challenging task. Towards this goal, in 2002, the pseudomorphic synthesis was introduced by Galarneau and co-workers [9,10,11,12,13]. The advantage of this method is that preformed, spherical silica gel particles with a non-ordered pore system can be used and transformed, in the presence of surfactant as a structure-directing agent, into ordered mesoporous materials like MCM-41, MCM-48 and MCM-50 by a dissolution-reconstruction process under preservation of the particle morphology. This so-called “pseudomorphic transformation” also allows obtaining MCM-41 materials with pore sizes from 6 to 9 nm by addition of trimethylbenzene to the synthesis mixture containing CTABr as surfactant [13].

Organo-modified spherical MCM-41 particles were obtained by post-transformation modification [14] or by addition of 3-(2-aminoethyl aminopropyl) trimethoxysilane to the reaction mixture during pseudomorphic transformation [15]. Lim *et al.* [16] and Martin *et al.* [10] used pseudomorphic transformation for a simultaneous introduction of Co and Al into the MCM-41 spheres for catalytic applications. Botella *et al.* [17] applied the pseudomorphic transformation for the preparation of MCM-41 nanoparticles with gold kernels.

A related approach was reported by Mokaya [18] who investigated the post-synthesis modification of mesoporous MCM-41 by a new synthesis mixture with MCM-41 as silica source to increase the pore size and the wall thickness of the parent MCM-41 material. The MCM-41 particles were preserved and underwent a morphological transformation of spherical shape into sheets and plates. Furthermore, Xia and Mokaya [19] prepared MCM-41-type materials by pseudomorphic transformation of HMS, MCM-48 and SBA-15.

Here, we report on the pseudomorphic transformation of a spherical silica gel using an aqueous alkyltrimethylammonium hydroxide solution instead of the hitherto mostly applied alkyltrimethylammonium bromide and NaOH solutions. Our approach, thus, combines the function of the base (mineralizer) and the surfactant (structure-directing agent, SDA) within one substance. This strategy was shown to be suitable for the transformation of pre-shaped pellets of a commercial zeolite Y to MCM-41 [20]. Recently, our approach was also applied by Patzsch and Schneider [21] for the transformation of amorphous silica microtubes into an MCM-41-based material. As the presence of sodium cations is known to disturb the formation of MCM-41-type materials [22], it was one aim of this study to compare the products of pseudomorphic transformation with CTAOH solution to those obtained with CTABr/NaOH solution as widely reported in the literature (vide supra).

The application of CTAOH together with CTABr allows the production of hierarchically structured pore systems by partial pseudomorphic transformation of shaped porous materials [23]. Thus, porous glasses with different shapes [23] or granules of commercial dealuminated zeolite Y [20] can be partially transformed to MCM-41 by using different amounts of CTABr/CTAOH at constant overall CTA concentration to control the degree of transformation. In most of the earlier studies, the synthesis time was applied to adjust the fraction of transformed starting material.

Often, an improved mass-transfer within the particle after transformation from a silica gel to MCM-41 or -48 was invoked [12,13]. Nevertheless, reports on the direct assessment of mass-transfer within the products of (partial) pseudomorphic transformation are still scarce. As recently shown by Adem *et al.* [24], PFG NMR spectroscopy is a powerful technique to study diffusion properties within nanoporous silica particles obtained from pseudomorphic transformation. While these authors describe the application of PFG NMR spectroscopy for characterizing the particles transformed at different degrees (obtained after different synthesis times), we focused here on application of PFG NMR diffusometry to obtain insight into the mechanism of the pseudomorphic transformation in the presence of different amounts of CTAOH solution. It was, thus, another goal of this study, to examine whether the use of the CTAOH instead of CTABr and NaOH exerts any influence on the diffusional properties within the pseudomorphically transformed materials. Moreover, we present complementary data from mercury intrusion to support the results obtained from PFG NMR spectroscopy measurements.

## 2. Experimental Section

### 2.1. Materials

Pseudomorphic transformation of the silica gel LiChrospher^®^ Si 60 (Merck) was carried out using an aqueous solution of CTAOH. This was prepared by ion exchange of an aqueous solution of CTABr (>98%, 71 g in 1000 mL of deionized water. Sigma-Aldrich, St. Louis, MI, USA) over the anion exchanger Amberjet 500 OH (Sigma-Aldrich, 100 g) for 24 h at room temperature under stirring. The final concentration of the resulting CTAOH solution was 0.195 mol/L. For the transformation, a mixture of 1 g of LiChrospher^®^ Si 60 and different volumes of the CTAOH solution (Table 1) was stirred for 1 h at room temperature and then, transferred to PTFE-bottles (100 mL) and kept for 24 h at 383 K under static conditions. The resulting solids were removed by filtration, washed with 100 mL deionized water, dried for 24 h at 383 K and calcined for 6 h at 813 K in an air atmosphere. Typically, 0.95 g of the transformed material was recovered.

**Table 1:** Volume of CTAOH solution V_CTAOH_ and molar composition of synthesis mixtures for pseudomorphic transformation of 1 g silica gel LiChrospher^®^ Si 60.

Sample	V_CTAOH_/cm^3^ g^−1^	SiO_2_	C_16_TMAOH	H_2_O
S1	9	1	0.042	30.0
S2	14	1	0.065	46.7
S3	19	1	0.088	63.3
S4	24	1	0.111	80.0
S5	29	1	0.135	96.7
S6	35	1	0.162	116.7
S7	42	1	0.195	140.0

Likewise, the transformation was performed with alkyltrimethylammonium hydroxide solutions with an alkyl chain length of C12, C14 and C18 (prepared from the bromides (Aldrich, >98%) by anion exchange as described above) for adjusting the mesopore width of the transformed products.

### 2.2. Characterization

Nitrogen sorption isotherms were recorded at 77 K on a micromeritics ASAP 2010 instrument. The mesopore diameter was calculated from the adsorption branch of the isotherms using the DFT model for slit or cylindrical pores. Specific BET surface areas were obtained from the linearized Brunauer-Emmett-Teller-Equation between p/p_0_ = 0.05 and 0.20. The total pore volumes were calculated from the isotherm point at p/p_0_ = 0.995. The degrees of transformation of the samples were calculated by dividing the MCM-41 mesopore volume obtained from the t-plot through the single point pore volume at p/p_0_ = 0.995. Mercury intrusion measurements were carried out on a Pascal 440 apparatus (ThermoFinnigan, San Jose, CA, USA). The samples were finely ground before the measurements to avoid pore-blocking effects. The cumulative pore volume at a given pressure represents the total volume of mercury taken up by the sample at the respective pressure. SEM pictures were obtained on a Philips ESEM XL 30 FEG microscope. The particle size distribution was determined on a CILAS 1064 instrument. Phase analysis of the samples was performed by powder X-ray diffraction (XRD, Siemens D 5000, Siemens Inc., Saarbrückenn, Germany) with Cu K_α_ radiation for the angle range of 2*θ* = 0.9°–10.0° with a step size of 0.05°.

### 2.3. PFG NMR Spectroscopy

The PFG NMR spectroscopy studies were done using a home-built spectrometer operating at 400 MHz for protons and equipped with a pulsed magnetic field gradient unit. For diffusion experiments, the 13-interval stimulated spin-echo pulse sequence was employed, which allows for minimizing undesirable effects due to internal magnetic field gradients [25]. With this technique, the process of molecular propagation in the direction of the applied magnetic field gradient is monitored during the observation time Δ [26]. In the experiments, the latter was varied between Δ = 6 ms and Δ = 200 ms in order to vary the length scale over which the diffusion process is registered. Other experimentally relevant parameters were: typical separation *τ* = 1 ms between 90° and 180° radio-frequency pulses in the 13-interval pulse sequence; the duration of the gradient pulses was *δ* = 400 µs. As a diffusant, n-heptane (Sigma-Aldrich, 99%) was used as obtained. The porous material under study, which was placed in NMR glass probes, was kept in contact with the vapour of n-heptane for 3 h at a pressure corresponding to 90% of the saturated vapour pressure for n-heptane. In this way, loading of only the mesopores by gas adsorption was ensured. Thereafter, the probes were sealed.

## 3. Results and Discussion

### 3.1. Materials

The pseudomorphic transformation of the amorphous silica gel LiChrospher^®^ Si 60 (main particle size: 12 µm, pore width: D_P_ = 6 nm) to the ordered mesoporous material MCM-41 was performed using an aqueous solution of CTAOH. Different degrees of transformation were obtained not, as reported in most of the earlier work, e.g., in [24], by interrupting the synthesis after different synthesis durations, but by using different volumes of the CTAOH solution per unit mass of the initial silica gel. Thus, the transformation stopped when the CTAOH supplied for dissolution of the initial silica gel and “re-precipitation” of the MCM-41 product was consumed. The point of interrupting the transformation was, therefore, more well-defined, easily controllable and less dependent on cooling rates involved when stopping the preparation after a certain time.

**Figure 1:**
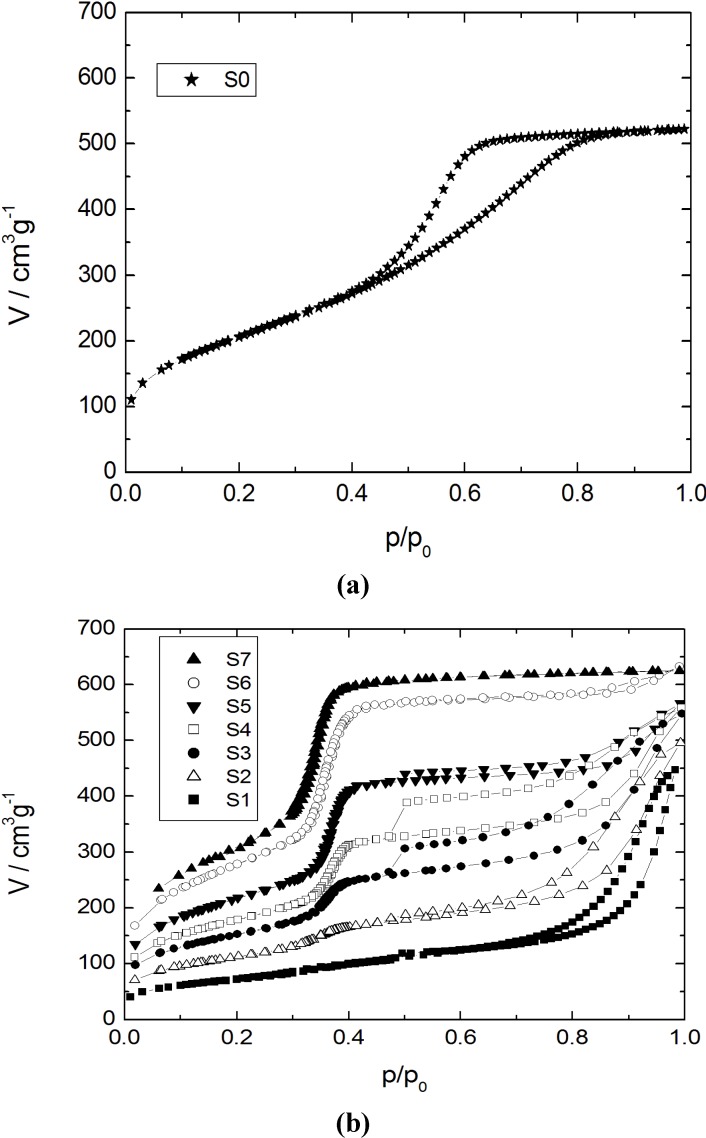
Nitrogen sorption isotherms at 77 K for (**a**) the initial silica gel S0; and (**b**) the transformation products S1–S7.

**Figure 2:**
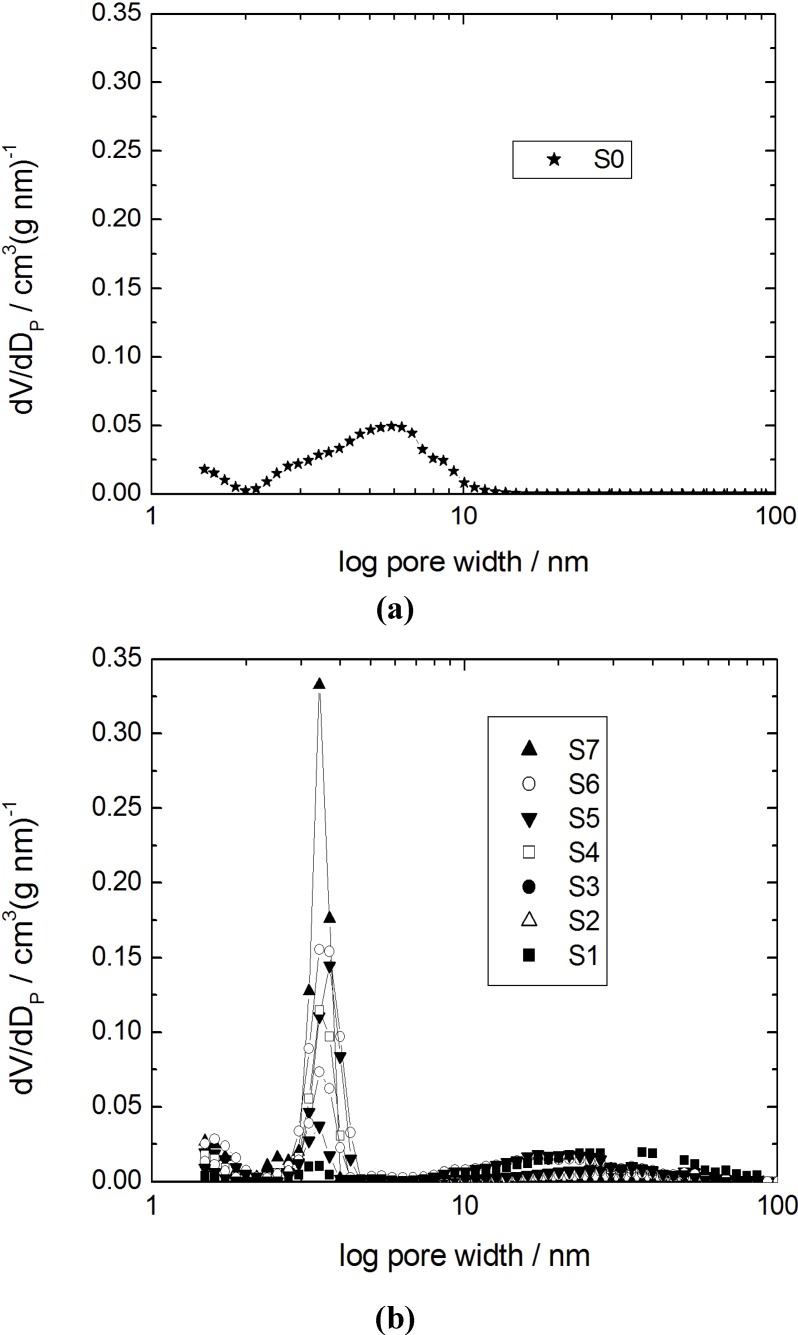
Pore width distributions calculated using the density functional theory (DFT) model (slit pore) for (**a**) the initial silica gel S0; and (**b**) the transformation products S1–S7.

The adsorption-desorption isotherms for nitrogen at 77 K and the corresponding pore width distribution functions for the initial silica gel and the products with different transformation degrees are shown in Figure 1 and Figure 2, respectively.

Specific surface areas and pore volumes of the initial silica gel and the products of partial and complete pseudomorphic transformation are given in Table 2. The isotherm for the parent silica gel (sample S0) shows a typical type IV isotherm for mesoporous and non-ordered materials with a broad pore width distribution. For the sample S1 with a transformation degree to MCM-41 of 10% only, the mesopore volume of the initial silica gel has completely disappeared giving rise to much larger mesopores with a broad width distribution in the range of 10–80 nm (Figure 2b) and a strongly reduced specific surface area from 735 m^2^ g^−1^ for S0 to 290 m^2^ g^−1^ for S1 (*cf.* Table 2).With an increasing amount of CTAOH solution, *i.e.*, with an increasing transformation degree, the shape of the N_2_ sorption isotherms of the transformation products changes dramatically (samples S3 to S5). In the region of relative pressure of about 0.37, a sharp increase of the adsorbed N_2_ volume is observed. This corresponds to the formation of the ordered mesoporous MCM-41-type material. As already reported by Martin *et al.* [10] and Yasmin and Müller [14], a type I hysteresis is observed for p/p_0_ = 0.5–0.9. This is caused by cavitation, when the condensed N_2_ desorbs from larger mesopores through the newly formed MCM-41 mesopores. From the pore width distributions of the samples S3 to S5 and considering the degree of transformation (*cf.* Table 2), it becomes clear that the content of the MCM-41 material continuously increases, while the volume of the larger mesopores decreases. In the case of sample S6, the type I hysteresis disappears and the rise of the N_2_ sorption isotherm in the region of p/p_0_ > 0.85 reveals that only some large mesopores remain. The isotherm and the pore width distribution of sample S7 show that an almost complete transformation of the initial silica gel into MCM-41-type material has occurred. The increase of the adsorbed volume at p/p_0_ = 0.37 is very sharp and only a slightly linear slope of the isotherm at higher relative pressures is seen. This is an indication of the absence of any large mesopores and corresponds to the low outer surface of the silica spheres.

These results already provide evidence that the first step in the pseudomorphic transformation of the silica gel beads of LiChrospher^®^ Si 60 is the dissolution of the parent silica, while the formation of the MCM-41-type silica phase occurs at a later stage within the course of the preparation. This is in contrast to the assumption of Galarneau *et al.* [9] that for a successful synthesis the same rates of silica dissolution and MCM-41 precipitation are needed.

**Figure 3:**
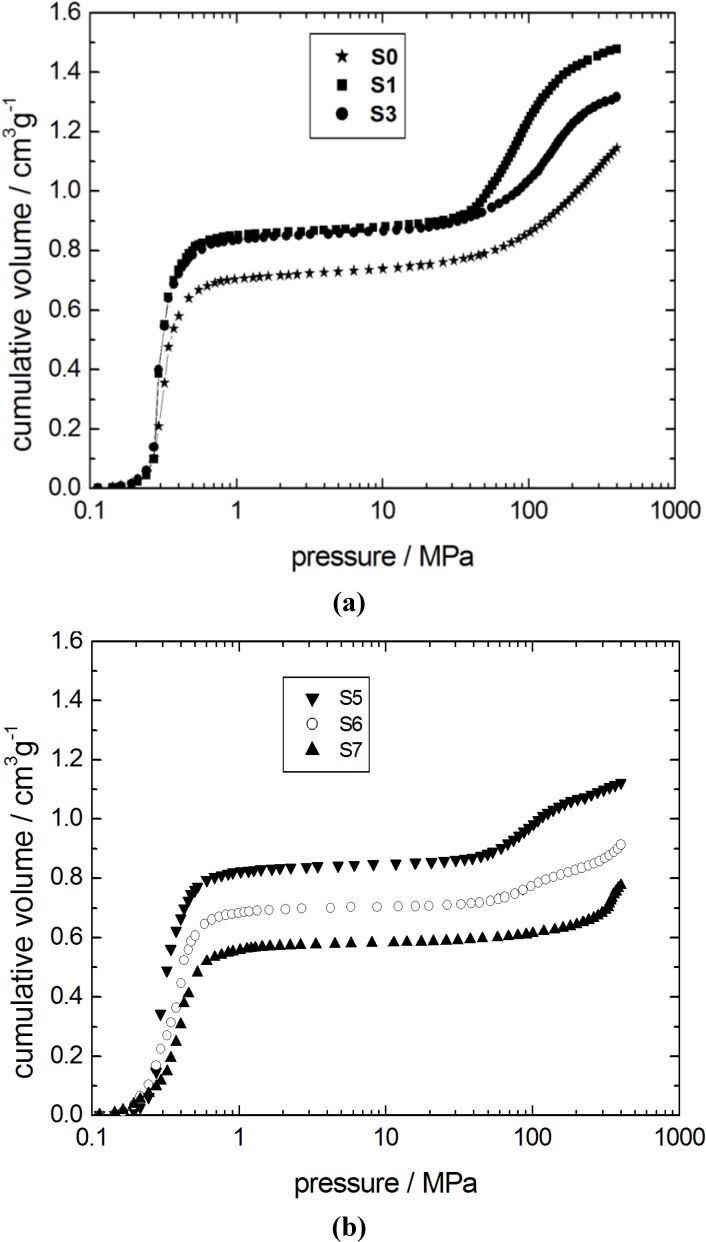
Mercury intrusion curves for the initial silica gel S0 and the transformation products (**a**) S1, S3; and (**b**) S5–S7.

The findings of the N_2_ adsorption isotherms are in good agreement with the results obtained from mercury intrusion porosimetry (Figure 3). The mercury intrusion curve of the parent silica gel S0 is characterized by two independent steps. The first step at low pressures (0.3–0.5 MPa) is generated by the interparticle space between the silica gel spheres. The mesopores inside the spheres are filled in the second step at higher pressures, *i.e.*, at 100–400 MPa. The dissolution of the parent silica network is apparent from the shift of the second step towards lower pressures from sample S0 to S1. This is, again, an indication for an increasing pore width of the transforming silica material.

**Table 2:** Specific surface area A_BET_, specific pore volume *V*_P_, obtained from nitrogen sorption (index: N_2_) and mercury intrusion (index: Hg), porosity and degree of transformation (DT).

Sample	A_BET_/m^2^ g^−1^	V_P, N2_/cm^3^ g^−1^	V_P, Hg_/cm^3^ g^−1^	DT/%
S0	735	0.80	1.14	0
S1	290	0.70	1.58	10
S2	415	0.77	1.48	22
S3	540	0.82	1.31	37
S4	642	0.89	1.22	50
S5	693	0.84	1.12	67
S6	860	0.94	0.91	91
S7	1050	1.04	0.77	100

Three independent steps are observed in the mercury intrusion curves of the samples S5 and S6 (Figure 3). The first step at low pressures does not change significantly with respect to the initial silica gel. In contrast, the amount of mercury intruded during the second step is reduced with progressing transformation from sample S3 to S6. Furthermore, an additional step at very high pressures above 300 MPa is observed. This is clear indication of the progressive transformation of the initially amorphous material into the ordered mesoporous MCM-41 phase.

**Figure 4:**
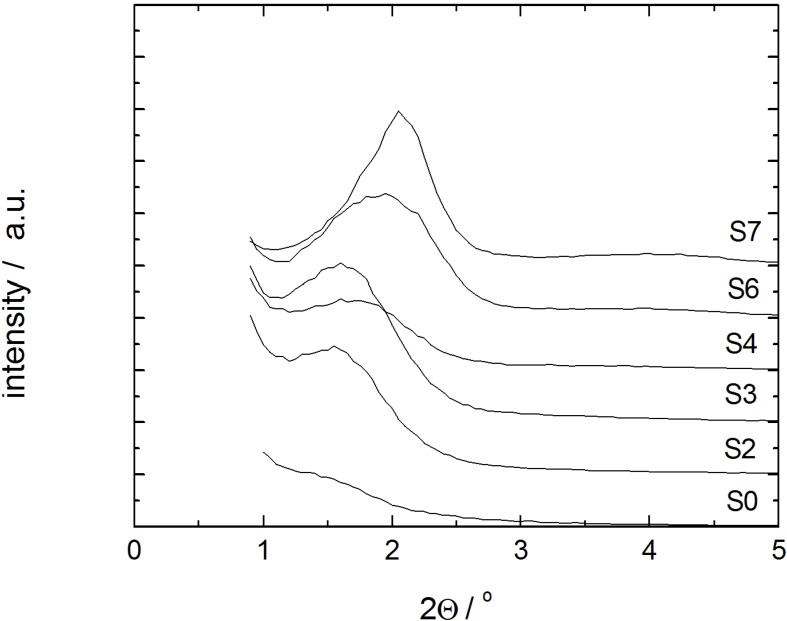
Powder XRD patterns of the initial silica gel S0 and the transformation products S2–S4, S6 and S7 (individual graphs are shifted by 1000/a.u.).

The second step of mercury intrusion has completely disappeared in sample S7. Simultaneously, the high pressure intrusion step between 300 and 400 MPa is now more pronounced. The unmodified structural mesopores typical for MCM-41 are filled with mercury in this pressure range [22]. Furthermore, a decrease of the interparticle porosity is observed as evident from the decreasing mercury volume penetrated in the low pressure step (0.3–0.5 MPa) of sample S7. The mercury intrusion curve of sample S7, thus, indicates the essentially complete transformation of the parent silica into MCM-41 material. The X-ray diffraction patterns of samples with different degrees of transformation are given in Figure 4. In case of the samples S3, S4 and S6, a broad diffraction peak indicative of MCM-41 with poor long-range order appears. Only for sample S7, this diffraction peak, indexed as 100, becomes completely transformed.

**Figure 5:**
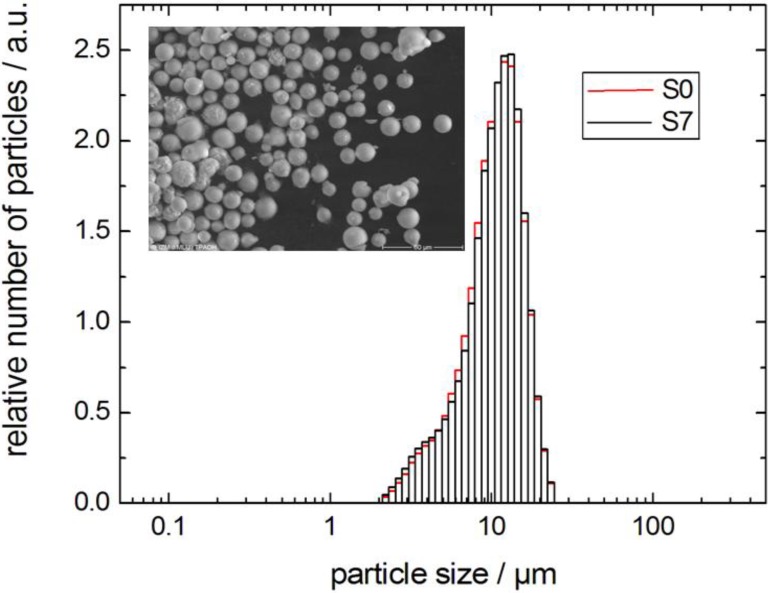
Particle size distribution of LiChrospher^®^ Si 60 (sample S0) and MCM-41 (sample S7) from pseudomorphic transformation and SEM image (inset).

The environmental scanning electron microscopy (ESEM) picture of the sample S7 and the particle size distributions of samples S0 and S7 in Figure 5 show the preservation of the size and the morphology of the silica spheres during the pseudomorphic transformation. For some of the beads, small aggregates can be seen on the outer surface. The completely transformed material contains approximately 80% of the silica contained in the parent material. One reason for the loss of material is the dissolution of silica from the large mesopores and its transport into the liquid phase surrounding the silica spheres. A second cause is the lower density of the MCM-41 material as compared to the parent silica gel. As a consequence, only part of the dissolved silica can be re-precipitated within the large mesopores of the spheres during the transformation process. This is in accordance with the results of Galarneau *et al.* [10] after which the pore volume of the parent silica determines how the precipitating MCM-41 material is accommodated within the particle.

**Figure 6:**
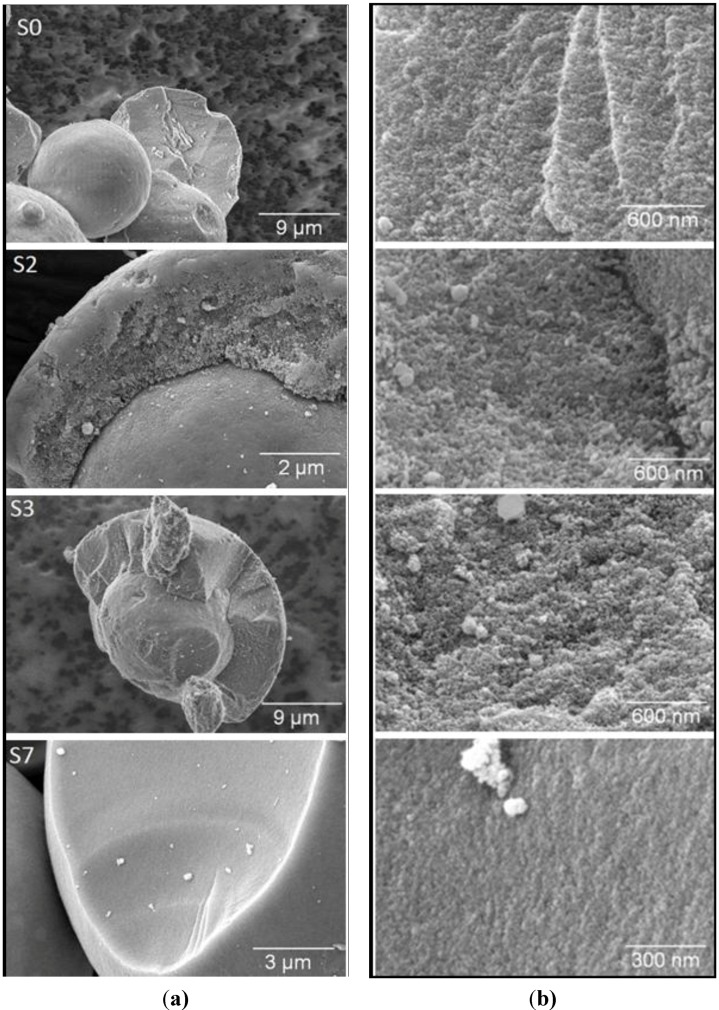
SEM images of (**a**) broken spheres; and (**b**) the view of the broken surface for the initial silica gel S0 and the transformation products S2, S3 and S7.

In Figure 6, ESEM pictures of the samples S0, S2, S3 and S7 are shown. On the left side, carefully broken pieces of the spheres are displayed in the micrometer scale. The pictures on the right side present a view of the broken area with higher magnification. For the parent silica gel (sample S0), only a rough surface is visible. In the case of the samples S2 and S3, the pictures seem to imply that the transformation has only occurred in an outer shell of the spheres. However, since the original pore structure could not be detected in the samples by N_2_ sorption or mercury intrusion, remainders of the initial silica gel can be excluded. Nevertheless, an inhomogeneous progress of the transformation over the particle cannot be ruled out. The pictures on the right side show a higher degree of porosity for the partially transformed samples S2 and S3 compared to the parent sample S0. In the case of the completely transformed material S7, only the flat surface and some larger pores can be seen. The homogeneity of the broken surface can be taken as evidence for the completion of the transformation process.

In the following, it was tested whether the transformation with alkyltrimethylammonium hydroxides can also be performed with different lengths of the alkyl chain. With the goal to adjust the pore width of the resulting MCM-41 product, alkyltrimethylammonium hydroxides with 12, 14 and 18 carbon atoms in the alky chain (denoted as C_12_TMAOH, C_14_TMAOH and C_18_TMAOH, respectively) were used during the transformation. Specific surface area, pore volume, and pore width from nitrogen sorption are reported in Table 3.

**Figure 7:**
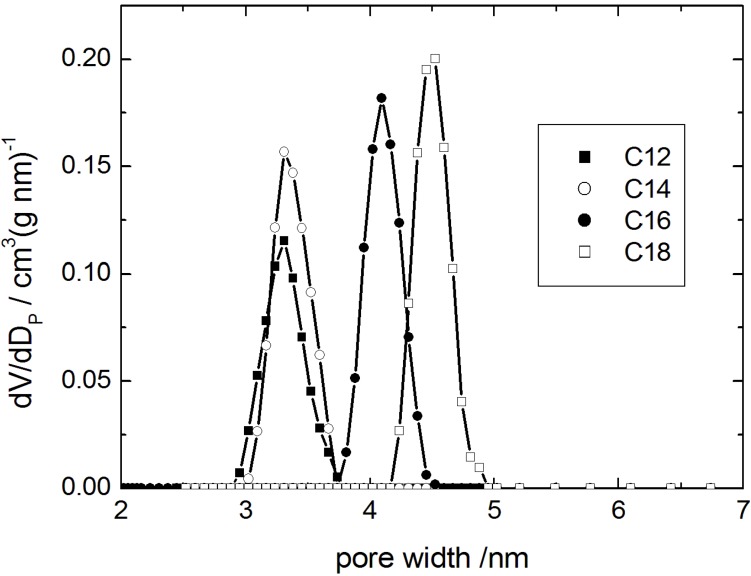
Pore width distribution calculated using the DFT model (cylindrical pore) for transformation products obtained using alkyltrimethylammonium-based surfactants with alkyl chain lengths of C12, C14, C16 and C18.

The pore width distribution functions obtained by the DFT-cylinder model are given in Figure 7 (for the N_2_ sorption isotherms see ESI). The transformation products are characterized by a high specific pore volume of the resulting MCM-41-type materials and a sharp pore width distribution providing proof for a high degree of order. Especially, the specific pore volume is higher than that reported for similar materials in [11,12,14,15,19,24,27], e.g., 1.04 (CTAOH, Table 3) *vs.* 0.90 cm^3^ g^−1^ (CTABr/NaOH [24]). These results clearly show that the transformation in the presence of alkyltrimethylammonium hydroxides leads to materials with improved order than when the transformation is carried out with the alkyltrimethylammonium halides and alkali hydroxides.

**Table 3:** Specific surface area A_BET_, specific pore volume V_P_, and pore width D_P_ from nitrogen sorption for the transformation products obtained using alkyltrimethylammonium-based surfactants with alkyl chain lengths of C12, C14, C16 and C18.

Surfactant	A_BET_/m^2^ g^−1^	V_P_/cm^3^ g^−1^	D_P_/nm
C_12_TMAOH	954	0.68	3.2
C_14_TMAOH	1177	1.06	3.3
C_16_TMAOH	1050	1.04	4.1
C_18_TMAOH	1018	1.10	4.5

### 3.2. Diffusion Studies

The above mentioned results from N_2_ sorption and mercury intrusion have clearly revealed structural changes during the pseudomorphic transformation of the initial silica gel into MCM-41 spheres. Moreover, the observation of the cavitation phenomena during desorption indicates that some fraction of the larger pores in the intermediate materials is connected to the external gas phase via the MCM-41 mesopores. The question, however, of how the domains of the growing MCM-41 are spatially arranged and how these evolve during the transformation has not yet been clarified. For instance, the ESEM pictures in Figure 6 implied a spatially separated formation of transformation products. To answer these questions and to compare the results obtained here using CTAOH solution for the transformation with those obtained with cetyltrimethylammonium halide and sodium hydroxide [24], we performed studies of the transport properties of fluids in the pore space of the materials used with the help of the PFG NMR technique.

**Figure 8:**
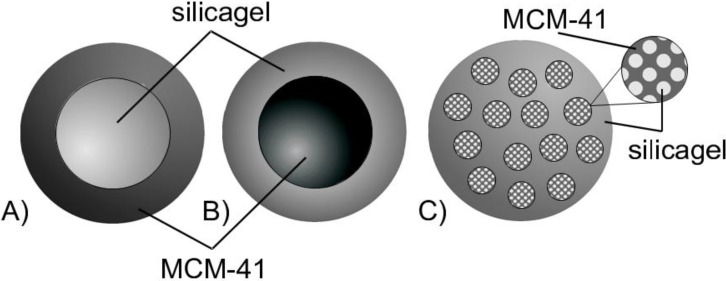
Schematic representation of products from partial pseudomorphic transformation showing the possible spatial arrangements of regions comprising initial (silica gel) and transformed (MCM-41) material (**A**,**B**) core-shell models; (**C**) homogeneous distribution model.

Taking this route, we have relied on an extensively documented study reporting on structure-dynamics relationships for fluids in pore spaces. Planning this type of experiment, in our particular case, we anticipated that if the regions of the parent material and the regions transformed to MCM-41 will be spatially separated on the micrometer length-scale, *i.e.*, that of the silica gel beads, we should observe two distinct diffusion processes. This would be due to different pore sizes in each of these regions leading to differing diffusivities of probe molecules. Such possible configurations are schematically shown in Figure 8.

Notably, the first one, which may be referred to as core-shell model with MCM-41 material forming the shell (Figure 8A), is consistent with the N_2_ sorption data revealing a cavitation effect. On the other hand, if the newly formed regions will be homogeneously dispersed within the parent silica gel (see Figure 8C), a particular dependence of the effective diffusivity on the transformation level may yield information on their spatial arrangement [28,29]. The case of the transformation progressing from the inside to the outside of the silica sphere resulting in a core-shell model with MCM-41 material forming the core (Figure 8B) is also taken into consideration.

**Figure 9:**
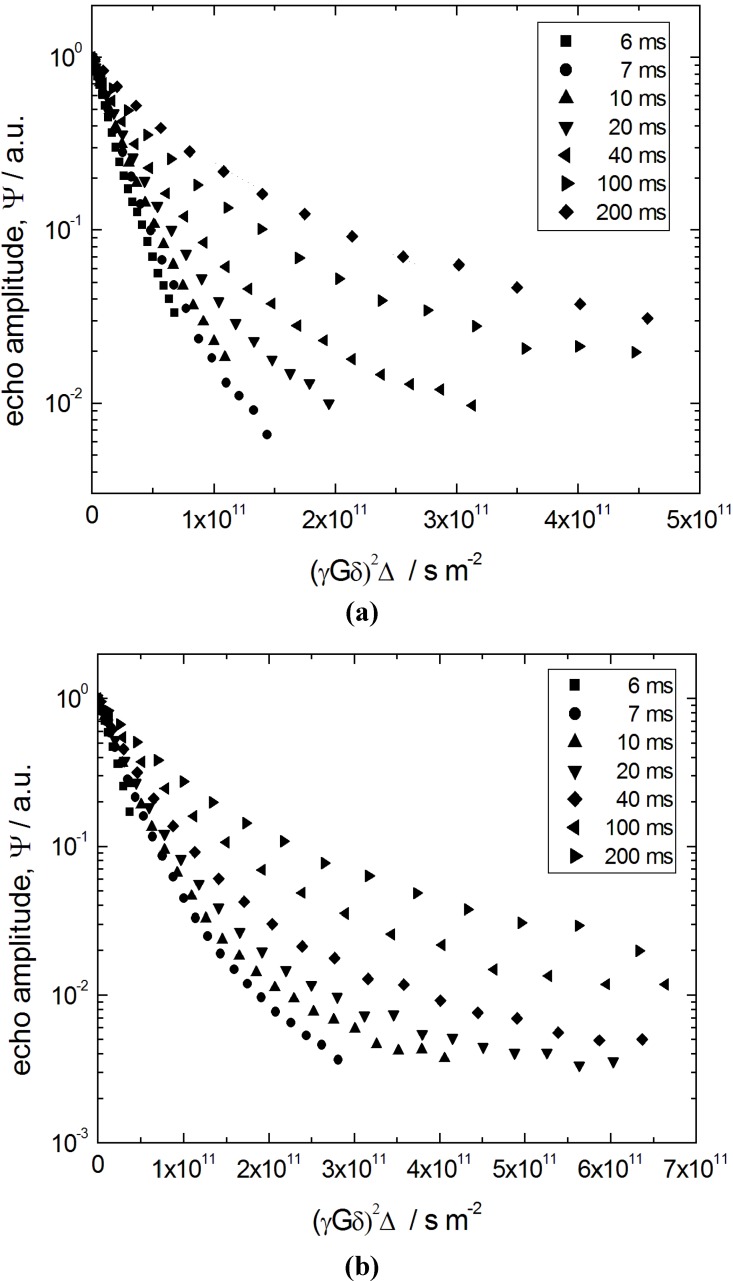
Spin-echo diffusion attenuation curves at different diffusion times of Δ = 6, 7, 10, 20, 40, 100 and 200 ms for (**a**) n-heptane in the initial silica gel (S0) obtained; and (**b**) the completely transformed material S7.

Figure 9 shows the primary quantity obtained using PFG NMR, namely spin-echo diffusion attenuations *ψ* (*q*, Δ), where *q* ≡ *γδG* denotes the so-called wave number (*γ* is the nuclear gyromagnetic ratio) controlled in the experiments and Δ is the diffusion time (also referred to as observation time) defined by the PFG NMR pulse sequence. The data in Figure 9 refer to n-heptane in two porous materials, the initial silica gel (S0) and the completely transformed (S7) ones. In the intermediate materials with different degrees of transformation, the diffusion attenuation functions are found to be qualitatively similar.

It is worth reminding here that, in all samples used, only the intra-particle pore space (*i.e.*, only the mesopores) was completely saturated by the capillary-condensed n-heptane by keeping the porous material in contact with the gas of n-heptane at a pressure of *P* = 0.9 *P_s_*, where *P_s_* is the saturated vapor pressure. Thus, no excess liquid existed in the space between the silica beads. This is in contrast to a similar study performed by Adem *et al.* [24], where n-hexane at partial pore loading of about 40% of the mesopore space has been used. In the latter case, a significant contribution of the mass transfer through the gaseous phases in the mesopores to the overall diffusivities is expected at room temperature. Indeed, exactly due to this reason, the data of Table 3 in [24] show the intrapore effective diffusivities exceeding that in the bulk liquid. Under these circumstances, due to a very complex relationship between a particular distribution of the capillary-condensed phase within the material and the resulting effective diffusivity [30,31], an accurate assessment of the structural details is difficult. Therefore, all measurements were performed in the present study at full pore loading to ensure that all parts of the pore system are probed under conditions of locally-identical (on the pore length scale) mobilities. Thus, n-heptane, a liquid with a low saturated vapour pressure, has further been used to suppress unwanted effects of molecular exchange with the gaseous phase between the beads.

The attenuation functions *ψ* (*q*, Δ) in Figure 9 reveal two important features. First of all, their shape deviates from the simple exponential function typical of normal diffusion processes. In fact, for a diffusion process characterized by a Gaussian propagator *ψ*(*q*, Δ) attenuates according to *ψ*(*q*, Δ) ∝ exp{− *q*^2^ 〈*r*^2^ (Δ)〉/6} = exp{− *q*^2^ Δ*D*}, where 〈*r*^2^ (Δ)〉 is the mean squared displacements and *D* is the diffusivity. Secondly, *ψ* (*q*, Δ) strongly depends on the observation time Δ. Importantly, the particularity of this dependence, namely an upward shift of the diffusion attenuation functions with increasing Δ, indicates increasing hindrance for the diffusion with increasing diffusion time. Formally, these two features may be interpreted as the existence of an apparent spectrum of the diffusivities, which shifts towards slower diffusivities with increasing diffusion time.

All the features revealed by the data of Figure 9 may uniformly be explained taking into account (i) a mean size of the particles of about 10 µm; (ii) a distribution of their size around the mean value; and (iii) a relatively low saturated vapor pressure *P_s_* of n-heptane. The latter leads to reflecting boundary conditions for the molecules at the particle boundaries, which may give rise to the restricted diffusion. Indeed, if during the diffusion times used in the experiment the molecules can displace by distances comparable to the particle size, upon reaching the boundary, they will be reflected back with a high probability. Thus, their mean square displacements 〈*r*^2^ (Δ)〉 acquired will be restricted by values of the order of *R*^2^ (more precisely to 6*R*^2^ /5 at infinitely long observation times), where *R* is the particle radius. Altogether, at short times, when the boundary effects are negligible, 〈*r*^2^ (Δ)〉 will grow linearly with time and, upon a certain transient, saturate at 6*R*^2^ /5 at long times. Thus, the conditions (i) and (iii) define the time scale on which the slopes of ln (*ψ*(*q*, Δ)) plotted versus *q*^2^ Δ will change with varying Δ [30,31]. The condition (ii) explains the observation of strong deviations of the diffusion attenuation functions from simple exponential forms due to a distribution of the particle or restriction sizes, leading to the respective distribution of the mean square displacements 〈*r*^2^ (Δ)〉 obtained for each particle [26].

In terms of diffusivities, which may now be referred to as apparent ones and defined via the Einstein relation as *D_app_* = 〈*r*^2^ (Δ)〉 /6Δ, at short times one obtains a constant diffusivity *D_app_* (Δ) = *D*_*p*0_, corresponding to the diffusivity which one would obtain in an infinitely large particle, which transits to *D_app_* (Δ) = *R*^2^ /5Δ at long times, when the molecules experience many collisions with the particle boundaries. Because there exists a distribution of the particle sizes, this results in the respective distribution of the apparent (time-dependent) diffusivities and, therefore, in the multi-exponential shape of *ψ* (*q*, Δ). Note that, at sufficiently short times Δ, the diffusivities do not depend on the particle size, yielding more exponential *ψ* (*q*, Δ). With increasing Δ, the effect of particle size becomes more pronounced giving rise to more complex shapes of *ψ* (*q*, Δ) in full accordance with the experimental results (Figure 9).

**Figure 10:**
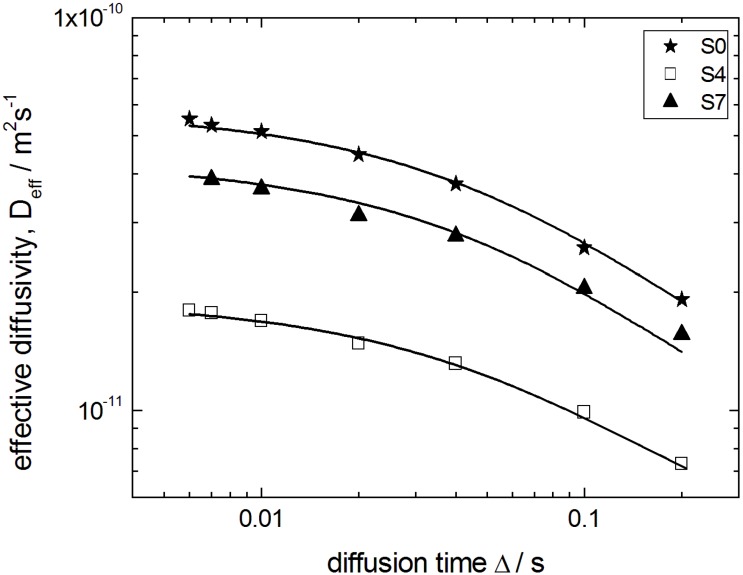
Effective diffusivities *D_eff_* of n-heptane in the initial silica gel S0 and the transformation products S4 and S7 as a function of the observation time Δ. Solid lines show best fits of Equation (1) to the data.

The occurrence of the time-dependent and multi-exponential character of the spin-echo attenuations on the time scale of PFG NMR due to non-trivial boundary conditions at the particle boundary complicates by far the extraction of the information of interest such as the genuine intraparticle diffusivity *D*_*p*0_ which is solely determined by the structure of the intraparticle pore space. To characterize this space, we analyze the effective diffusivities *D_eff_* (Δ), which are obtained from the slopes of ln (*ψ*(*q*, Δ)) versus *q*^2^ Δ in the low-*q* region. The thus obtained quantity *D_eff_* (Δ) is shown in Figure 10 for the initial silica gel S0, a partially (S4) and the completely transformed sample (S7). They may be considered as *D_app_* (Δ) averaged over all particles with different particle sizes *R*. Such an average results from the fact that in NMR the signal measured is a cumulative signal over all spins in the system under study.

The diffusivities in Figure 10 decrease with increasing Δ for all observation times studied from 6 ms to 200 ms, revealing the observation of the transient regime discussed above. This appears to be reasonable by estimating the molecular displacements $\sqrt{6D_{p0}\text{Δ}}$ from about 1 µm to *ca.* 8 µm during these observation times which are comparable to the particle sizes. For a rough estimate, *D*_*p*0_ has been approximated by the value of 5.0 × 10^−11^ m^2^ s^−1^ as obtained for the parent silica gel material at the shortest diffusion time. However, the values of *D*_*p*0_, which we are looking at, can be obtained more precisely based on a two-point Padé approximation [32].

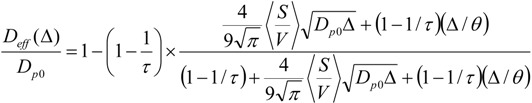
(1)

Equation (1) has been suggested as a helpful analytical expression connecting the short- and long-time limits for the time-dependent diffusivity. Here, *τ* is the tortuosity of the pore space; *θ* is the parameter having the dimension of time and describing the rate of the transition from the short- to the long-time limit; and < *S* / *V* > is the average surface-to-volume ratio of the particles. The latter has been calculated using the data of electron microscopy by compiling the particle size distribution histogram (*cf.* Figure 5) from the micrograph.

**Figure 11:**
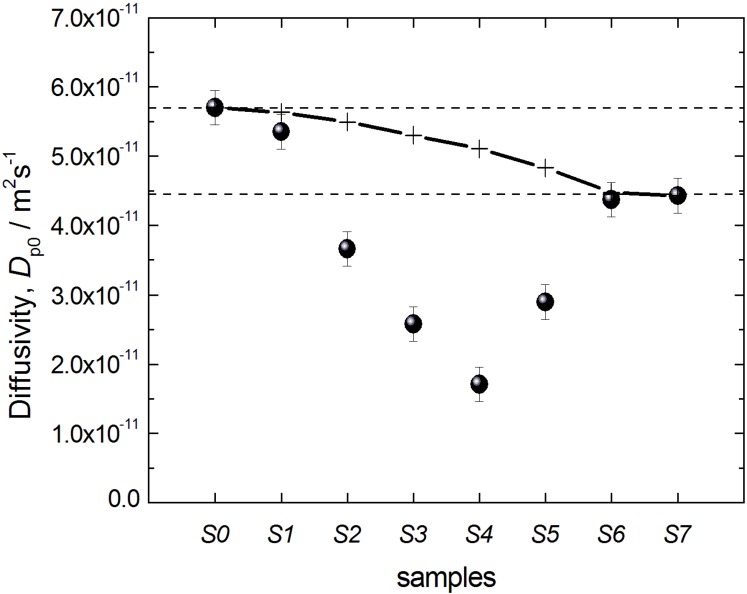
Effective diffusivities *D*_*p*0_ of n-heptane in the initial silica gel S0 and the transformation products S1–S7. The crosses and the solid line show the prediction of the two-region model (see text for explanations).

The best fits of Equation (1) to the experimental data are shown in Figure 10 by the solid line. Most importantly, in this way *D*_*p*0_ could be obtained with a sufficiently high accuracy. The thus obtained data on *D*_*p*0_ are shown in Figure 11 for all materials under study and reveal two notable features: (i) the diffusivities for the initial silica gel and the completely transformed MCM-41 material are found to be nearly identical; and (ii) the diffusivities for intermediate materials pass through a minimum with an increasing degree of transformation.

The real values of *D*_*p*0_ are determined by two factors. First of all, even in straight, channel-like pores, the effective diffusivities decrease with decreasing channel diameter, primarily due to interaction of fluid molecules with the pore walls [31,33,34]. Secondly, the tortuosity of the pore space also strongly affects the long-range diffusivity [35]. We anticipate that it is an interplay of these two effects that is responsible for the small difference between *D*_*p*0_ obtained for the initial silica gel S0 and completely transformed MCM-41-type material S7. Even if the pore diameters in the parent silica gel and the final MCM-41 differ notably, the final material presumably has a lower tortuosity, compensating thus the decrease of the diffusivity due to a decrease of the pore size. In fact, this is reasonable keeping in mind the rather disordered pore structure of the silica gel materials of the LiChrospher^®^ Si 60 type with various constrictions and cage-like pores. By forming MCM-41, the vast number of these pore-space elements is diminished, thus decreasing the transport resistance [36,37]. This explanation is also in-line with the results of Adem *et al.* [24] who have interpreted the increased tortuosity of the transformation product in terms of “stagnation zones” as opposed to straight channels.

The most interesting effect observed is the passage of the diffusivities through a minimum with progressing transformation. We anticipate, in line with the preceding discussion, that this is a result of the modification of the microstructure of the pore space. During the initial stages of the transformation, some regions containing MCM-41-type material are formed. Thus, some part of the parent silica gel becomes surrounded by the material with smaller pores, *i.e.*, by necks. This results in the decrease of the effective diffusivity due to an increasing cage effect. Notably, such a scenario is consistent with the finding of the cavitation phenomenon in gas adsorption. Further progress of the transformation first leads to the formation of an increasing number of domains of silica gel surrounded by MCM-41-containing regions, but after a certain stage, the volume of such regions will start to decrease, explaining the observation of the minimum in diffusivity (Figure 11).

Importantly, the data on *D*_*p*0_ in Figure 11 may also yield information on the spatial extension and arrangement of such domains. Indeed, from the possible options schematically depicted in Figure 8, the one shown in Figure 8B may be ruled out on the basis of gas adsorption measurements, because it cannot explain the observed cavitation. Case 8A, assuming that the transformation starts at the silica gel particle boundaries and the regions converted to MCM-41 grow towards the particle center, may, however, also be ruled out on the basis of the diffusion study results: Indeed, in case of a material composed of two macroscopically separated regions containing the initial silica gel (S0) and the MCM-41 (S7) only, the effective diffusivity obtained using PFG NMR spectroscopy will be given simply by the sum *D*_*p*0_ = *p*_*S*0_*D*_*p*0,*S*0_ + *p*_*S*7_*D*_*p*0,*S*7_, where *D*_*p*0,*S*0_ and *D*_*p*0,*S*7_ are the diffusivities in the two materials; and *p*_*S*0_ and *p*_*S*7_ are their relative pore-volume fractions (*p*_*S*0_ + *p*_*S*7_ = 1). On the basis of this equation, the expected behavior of the diffusivities measured is shown in Figure 11 by the solid line (and the crosses) and cannot reproduce the observed minimum of the diffusivity in the partially transformed materials. Therefore, we may conclude that these cage-like regions of the parent material have to be of dimensions smaller than the minimal molecular displacements registered in the PFG NMR experiments of the order of 1 µm and that they have to be homogeneously distributed over the particle, *i.e.*, silica sphere, volume. Thus, all effects leading to the decrease of the diffusivities at intermediate stages of the transformation are caused by structural changes on the length scale below 1 µm.

The final picture revealed by diffusion and gas sorption measurements, therefore, corresponds to a homogeneous distribution of regions from which the transformation progressively spreads over the initial silica particle as schematically depicted in Figure 8C. Notably, in this Figure we have shown that only a certain fraction of the silica gel is surrounded by MCM-41-containing regions. This is done to be consistent with the observation of the N_2_ desorption isotherms revealing that fluid only in some part of the pores are subject to cavitation-driven evaporation.

## 4. Conclusions

The pseudomorphic transformation of spherical silica gel (LiChrospher^®^ Si 60) was achieved by treatment with an aqueous solution of CTAOH. In comparison to the conventional synthesis using CTABr and sodium hydroxide, materials with comparable textural properties, but slightly improved specific pore volume and a narrower pore width distribution are obtained. Moreover, the transformation was completed within one day by using a higher amount of CTAOH solution with respect to the initial silica gel as opposed to several days needed for transformation with CTABr and sodium hydroxide. Partially transformed products were obtained by adjusting the ratio of CTABr solution to initial silica gel at constant synthesis time. This approach might be useful to prepare hierarchically structured materials by transforming a controllable fraction of shaped bodies of nanoporous materials into ordered mesoporous M41S phases, keeping the morphology of the starting material. Moreover, the pore width can be adjusted using alkyltrimethylammonium hydroxides with different carbon alkyl chain lengths as shown for 12–18 carbon atoms here.

A thorough analysis of the diffusion of n-heptane at full loading in the pore spaces of the initially, the partially and the completely transformed silica materials by PFG NMR spectroscopy was applied to obtain insight on the processes occurring during pseudomorphic transformation. Thus, the transformation is shown to occur via formation and subsequent growth of domains of <1μm containing MCM-41 which are homogeneously distributed over the volume of the silica spheres. Moreover, and in accordance with a recent literature study, the completely transformed silica spheres do not show improved diffusion properties over the initial silica gel as a result of the reduced pore size in combination with the lower tortuosity within the MCM-41 domains.
